# Youth and forecasting of sustainable development pillars: An adaptive neuro-fuzzy inference system approach

**DOI:** 10.1371/journal.pone.0218855

**Published:** 2019-06-25

**Authors:** Jasna Petković, Nataša Petrović, Ivana Dragović, Kristina Stanojević, Jelena Andreja Radaković, Tatjana Borojević, Mirjana Kljajić Borštnar

**Affiliations:** 1 University of Belgrade-Faculty of Organizational Sciences, Belgrade, Serbia; 2 University of Maribor-Faculty of Organizational Sciences, Kranj, Slovenia; International Maize and Wheat Improvement center (CIMMYT), MEXICO

## Abstract

Sustainable development goals are used as a guidance for strategies development on local, regional and national levels. The importance of including young people in this complex process is recognized in all relevant documents (i.e. Agenda 21), however it is not an easy task to elicit opinions and preferences from the youth. Furthermore, the assessment of the sustainable development goals itself presents a challenge for the noisy data and nonlinear relationships in data. Popular approach is fuzzy set models where expert knowledge is presented with comprehensible rules; however expert knowledge elicitation takes a long time too. Several studies proposed an adaptive neuro-fuzzy inference system approach that combines the fuzzy set theory to model expert knowledge with neural networks for inferring rules and membership functions from data to assess the sustainable development performance. We base our assumptions that ANFIS can be used to predict the importance of sustainable development pillars from the demographic data of young people. For this purpose, we have conducted an online survey on sustainable development goals opinions and importance of young people in Serbia. The sample of 386 respondents has been split into a training sample of 300 instances (to generate membership functions and fuzzy rules) and a testing sample of 86 instances to predict the importance of the three pillars. We have conducted a trace-driven simulation test to validate the results of the proposed ANFIS model. Results of the study provided insights into how the young people in Serbia assess the importance of sustainable development goals. Secondly, the results suggest that ANFIS can be applied to predict values of importance of the three sustainable development pillars with the relative error of Rel Err < 5%. It must be noted that the considered model could be further improved by using training samples with more data.

## Introduction

“*Today’s generation of young people is the largest in history*. *Over 3 billion people–nearly half of the world’s population–are under the age of 25*. *Almost 90% of all young people live in developing countries*. *Young people are a valuable asset to their countries and investing in them brings tremendous social and economic benefits*. *(…) It is crucial that we engage the young decision makers of tomorrow in the development decisions of today*.”

(Nemat Shafik, Permanent Secretary—Department for International Development)

Ever since the report of World Commission on Environment and Development: Our Common Future as urgent call by the General Assembly of the United Nations and “a global agenda for change” was published in 1987, the concept of sustainable development (SD) has gradually become a globally recognized development strategy of all nations [1]. On the other hand, today’s society, all communities and individuals are unfortunately facing numerous problems, contradictions, risks, and uncertainties related to further global SD, where it is stressed that the role of young people must be one of the main and necessary factors. The reason for this is to be recognized in the fact that young people represent the necessary link not only to establishing SD, but also to improving the quality of global and national societies’ functioning. It should be added here that the young people embody present and future of every society, and represent the source of innovations and the driving force of every development, including the SD [2]. The young participate in attaining the sustainability and goals of the sustainable development (SDGs) presented in the Agenda 21 [3], with an accent on their contribution in accomplishing the openness, participation and democracy [4–11].

Speaking of the young, it should be pointed out that various definitions of this term are being used in practice, and the same stands for various, mostly upper limit of their age, which is related to country’s local circumstances, economic condition and cultural characteristics. However, we can draw a conclusion that they are between 14 and 30-year old. When it comes to Serbia, those between 15 and 30 years of age [12, 13] fall under the category of young people, and they count around million and a half, which represents 20 percent of the total number of population of Serbia [2, 13, 14]. These data correspond with the overall number of young people worldwide. Interesting forecasts say that the population of young people will continue to grow, so that, for example, about 1.9 billion are projected to turn 15 between 2015 and 2030 [15].

There is a relatively small number of scientific papers on the subject of youth and the SD, and their attitudes and perception about SDGs, especially in Serbia [2, 12, 14, 16]. The involvement of youth in the policy making is scarce [12], on the other hand it is clear that it is very important [3, 12, 13 15]. In order to gain insights into young people’s assessment of the 17 SDGs in terms of their importance and priority in further SD of Serbia, we have conducted an online survey. The second goal was to test the possibility of application of Adaptive Neuro-Fuzzy Interference System (ANFIS) approach in forecasting youth assessment of the three pillars of SD–economic, environmental and social using demographic data only. The forecasted importance assessment of SDGs could provide valuable insights for evaluation of current implementation progress across the SDGs agenda as well as “level of change needed varies widely across regions and countries” [17].

Our research included 386 young people aged from 15 to 30 years old. We calculated the representative sample size of Serbian youth population by the online sample size calculator [18] taking into consideration the total number of young people in Serbia [19]. For the purpose of our research we used an on-line questionnaire that consisted of 28 questions in total. An analysis of the questionnaire was carried out using the SPSS 24 software package. The results obtained for marks given to importance of individual SDGs have then been categorized into adequate SD pillar.

The use of various models (AHP, fuzzy set, AI) have become increasingly important in addressing complex SD issues [20–22]. In our research we have chosen to apply the ANFIS model to forecast the youth assessment of the three pillars of SD, based on the obtained evaluation of importance of certain pillars. The importance of the SD pillars as perceived by the young people in Serbia, is a necessary input for more adequate use of the National Strategy of Sustainable Development of the Republic of Serbia on both the national and local level. This is explicated in the strategy, which strictly stresses the priority “that is being given to young people for creating conditions for gradual transition towards sustainable life styles in the Republic of Serbia, which are present and future pillars of sustainable development” [23]. We have chosen the ANFIS method for its “efficiency and its potential to capture a large amount of nonlinear and noisy data” [24]. Methods of artificial intelligence (including ANFIS) have been so far used for certain aspects of SD [25–32], but never for forecasting of all three pillars of SD in the case of young people, so the contribution of this paper is also recognized in the expansion of the body of knowledge that refers to the forecast of sustainability and the very application of ANFIS model.

The objective of this study was to examine the possibility of application of ANFIS model in forecasting of the three SD pillars in the case of young people of Serbia. ANFIS has been selected having in mind that methods of artificial intelligence play an increasingly important role in different areas of sustainability [30–32]. Consequently, our research has the intention to enable a framework of SD pillars prioritization which could provide grounds for effective and efficient implementations of sustainability objectives and SDGs based on adequate youth participation.

The paper is structured in following sections. In Section 2 we outline theoretical background with literature review on SD, SDGs, the role of youth in SD, and Adaptive Neuro-Fuzzy Inference System (ANFIS). In Section 3 we present the materials and methods that have been applied in the research with emphasizes on data analysis, and forecasting of SD pillars using the proposed ANFIS model. We present and discuss the result of the survey and ANFIS model forecasts in Section 4, and finally in Section 5 we draw conclusions.

## Theoretical background

### Sustainable development and the role of youth

The most commonly used definition of sustainable development was given by Lester Brown, the founder of the Worldwatch Institute. It has been quoted in the report entitled “Our Common Future” and it says: “Sustainable development is development that meets the needs of the present without compromising the ability of future generations to meet their own needs” [1].

The World Summit on Sustainable Development took place in Johannesburg in August, 2002 [33]. At this Summit, all member states have agreed to start producing and adopting national strategies of SD in as short period of time as possible. This was the last time that new dimensions were added to the definition on SD–namely, in addition to environmental protection it now included both economic and social goals. It should also be stressed that the theoretical literature discussed SD in depth, as this concept was set as a goal of economic and social development. This was agreed not only by United Nations agencies, but also by various local governments and private-sector agents–not to forget the Agenda 21 [1]. SD has at least three dimensions i.e. pillars: environmental (considering degradation of natural resources necessary for human use), social (indicating the unequal distribution of wealth and poverty), and economic (contending that any development meant to achieve sustainability needs to manage different capital flows) [34–36].

These three core elements of SD [4] gained even more credits [37]:

- Economic: A system that is sustainable in economic terms must be adept in producing goods and services in continuity, at the same time maintaining controllable levels of government and external debt.
- Environmental: A steady resource base must be kept by an environmentally sustainable system, this being done by not exhausting renewable resource systems or environmental sink functions.
- Social: A socially sustainable system must fairly distribute and adequately provision social services such as health and education, gender equity, and political liability and participation.

Today’s imperative is to nourish SD. New Global Sustainable Development Agenda aims to stop poverty, encourage prosperity and advocate people’s welfare while engaged in preserving the environment by 2030. The General Assembly of United Nations ratified the new resolution on 25 September 2015, which is actually a New Sustainable Development Agenda for the period between 2015–2030: Transforming our world: the 2013 Agenda for Sustainable Development. “The 17 sustainable development goals and 169 objectives that are being communicated today exhibit the range and ambition of this universal Agenda. They will continue to build on the Millennium Development Goals and attain what was not achieved. They aim at realizing the elementary human rights and to attain gender equality together with women empowerment. They are indivisible as they integrate the three dimensions of sustainable development: the social, economic, and environmental” [38].

SDGs are given in Table 1.

**Table 1:** SDGs [38].

Order	Goals
1.	End poverty in all its forms everywhere.
2.	End hunger, achieve food security and improved nutrition and promote sustainable agriculture.
3.	Ensure healthy lives and promote well-being for all ages.
4.	Ensure inclusive and quality education for all and promote lifelong learning.
5.	Achieve gender equality and empower all women and girls.
6.	Ensure access to water and sanitation for all.
7.	Ensure access to affordable, reliable, sustainable and modern energy for all.
8.	Promote inclusive and sustainable economic growth, employment and decent work for all.
9.	Build resilient infrastructure, promote sustainable industrialization and foster innovation.
10.	Reduce inequality within and among countries.
11.	Make cities inclusive, safe, resilient and sustainable.
12.	Ensure sustainable consumption and production patterns.
13.	Take urgent action to combat climate change and its impacts.
14.	Conserve and sustainably use the oceans, seas and marine resources.
15.	Sustainably manage forests, combat desertification, halt and reverse land degradation, halt biodiversity loss.
16.	Promote just, peaceful and inclusive societies.
17.	Revitalize the global partnership for sustainable development.

The Sustainable Development Goals (SDGs) deliver a vision, at the same time comprising positive and viable agenda for protecting both people and the Planet we live on. It’s a promise of a brighter future for our generation, and those generations that are yet to come. All the participants will get to walk the path of value creation by acknowledging that the benefits of SD must be combined with new partnerships and alliances. The truth is that the SDGs are in position to deliver value to a wide range of stakeholders: they bring positive transformational power to communities; Governments experience a fast-paced advancement; corporations accomplish even higher levels of efficiency and sustainability; both individuals and society enjoy the increase in the quality of life [39].

Further on, at the 2nd United Nations Conference on Environment and Development–UNCED the recommendations of the Bruntdland Commission were adopted. One of the most important results of the Summit was the adoption of Agenda 21 “which offered a practical approach to applying sustainable development policies at the local and national level, and the Rio Declaration on Environment and Development” [39].

Agenda 21 consists of four sections [3]:

- Section I–Social and economic dimensions.
- Section II–Conservation and Management of Resources for Development.
- Section III–Strengthening the Role of Major Groups.
- Section IV—Means of Implementation.

The role and the position of the young in SD has been described and given in detail in paragraphs 25.1–25.11 within the Section III, especially having in mind that the young people make about 30 percent of world population, and their participation in questions that refer to the environment and decision making, as well as to program implementation, represents a key component for long-term success of the Agenda 21 itself [3].

The program area of Agenda 21 that relates to the young emphasizes the promotion of the role of young people and their active engagement in environment protection and promotion of economic and social development, i.e. all three pillars of the SD. Because of that, ground for action has been given in paragraphs 25.2 and 25.3 [3]:

- It is an imperative for the young people from all over the world to actively participate in all relevant levels of the decision making process having in mind that those decisions affect not only their present lives, but their future as well. It should be added here that the intellectual contribution of young people and the possibility of giving support represent a unique perspective that should be also taken into consideration.
- Numerous actions and recommendations of the international community are advocated in order to provide possibility for the young to have steady and healthy future that includes the quality of the environment, improved living standards, and making it possible for them to get education and find jobs, whereas these questions need to be underlined in all development plans.

### Adaptive Neuro-Fuzzy Inference System (ANFIS)

The artificial neural network was defined as a system that consists of a large number of highly connected simple elements of processing that work parallel with each other. Every element has its own local memory in which it stores data that needs processing [40]. Neural networks allow for intelligent processing without previously defined model or algorithm, but based on data on system behavior.

The Fuzzy logic theory was proposed by Zadeh [41] in order to describe complex system, after which it became popular and effectively used for problem solving, mainly on control processes. One of the most important applications of the fuzzy logic is fuzzy inference system where the decision making is performed based on a set of rules. Fuzzy inference system consists of if-then rules that are understandable since they are written in a way that relates to people, but the parameters don’t have an option of setting based on past data. Just the opposite, a neural network is able to learn from the setting (input-output pairs), organize internal structure, and adapt to it interactively.

Adaptive Neuro-Fuzzy Inference System (ANFIS) is the most commonly used neuro-fuzzy system [41] and it was introduced by Jang [42], based on two approaches: artificial neural networks (ANN) and fuzzy inference systems (FIS) [43, 44] in order to build a system that uses the advantages of both, neural networks and fuzzy logic. ANFIS safeguards the interpretability through if-then rules together with the adaptability, because the neural network optimizes the membership functions of fuzzy rules based on the input-output data that describe the system behavior. In other words, neuro-fuzzy systems keep the transparency of the fuzzy system and the ability of learning which is inherent to neural networks.

ANFIS is good at learning, designing, expensing, and classifying. It allows for the extraction of fuzzy rules from expert knowledge or numerical data, in this way constructing a rule foundation. In addition, it can harmonize the complex conversion of human intelligence to fuzzy systems. The downside of the ANFIS predicting model is quite time-consuming training structure and method of determining parameters, especially when the number of input parameters is large. In addition, one of the main features of this system is its adaptability. Once the structure is established (number and the initial shape of the parameters of membership functions as well as operators of the rules) the system will set optimal values of parameters. The setting is performed in accordance with a training set based on gradient procedure.

The description of layers has been taken over from Jang [42], with the only difference that the description of system will use the shape and value of functions implemented for the purpose of this paper.

*Layer 1*: Each node of the first layer forwards values to the next layer.
*Layer 2*: It calculates the degree to which an input element *x*_*i*_ correlates to the linguistic label *A*_*i*_ (low, high…), in other words, the degree to which *x*_*i*_ satisfies the property of it. Here we used a bell-shaped function for the membership functions. Jang’s model supports the bell-shaped function with the maximal value in 1 and minimal value in 0:
  $$\mu_{Ai}\left(x\right)=\frac{1}{1+{\left[{\left(\frac{x-c_{i}}{a_{i}}\right)}^{2}\right]}^{b_{i}}}$$ (1)
  where {*a*_*i*_, *b*_*i*_, *c*_*i*_} represent a set of parameters that refer to the rule premise which needs to be optimized through the learning process.
*Layer 3*: every node of this layer corresponds to a separate rule. The task of each node is to multiply certain input signals (conjunction is implemented through products, i.e. the values of membership functions of the elements that are part of a given rule are being multiplied).
  $$w_{i}=\mu_{Ai}\left(x\right)\times \mu_{\prime Bi}\left(x\right),\;i=1,2,3\ldots$$ (2)
  The input is actually falsified values, while the output from every node represents the total strength of the rule and it is being forwarded to the next layer.
*Layer 4*: Task of this layer is to perform calculations that lead us to a normalized equation of an *i*^*th*^ rule.
  $$\bar{w_{i}}=\frac{w_{i}}{w_{1}+w_{2}},\;i=1,2,3\ldots$$ (3)
  The relative contribution, as a relation between the strength of the *i*^*th*^ rule against the sum of the strengths of all other rules, is being calculated for every node.
*Layer 5*: Every node *i* of this layer is a square-shaped node that performs the following function:
  $$0_{i}^{4}=\bar{\omega_{i}}f=\bar{\omega_{i}}\left(p_{i}x_{1}+q_{i}x_{2}+r_{i}\right),\;i=1,2,3\ldots$$ (4)
  where ω_*i*_ is the output from the previous layer 3, while *p*_*i*_*q*_*i*_ and *r*_*i*_ are the set of parameters that refer to the conclusion. The values *x*_*1*_ and *x*_*2*_ are the initial input values.

### ANFIS method application in SD assessment

SD is inherently a complex decision-making process with multiple interrelated variables, nonlinear and noisy data [32]. The SDGs serve as guidance for policy development, which vary according to the demographic, cultural, environmental, economic factors, and level of decision-making (national, regional, local) [38]. Various sustainability performance indexes are used to evaluate and benchmark the effectiveness policies and for future planning. For this purpose, various methods have been applied to model the SD and sustainability performance assessment, from multi-criteria decision-making methods to machine learning methods [20, 21, 22, 27, 28, 29, 43–45]. In the past years the ANFIS method had yielded favourable results compared to other methods [30–32, 46]. On a case of countries SD performance assessment authors provide evidence that the membership functions modelled by the ANFIS method can effectively address the drawbacks of fuzzy logic models in inferring membership functions from the past data [32]. By learning membership functions directly from 128 training examples of the SAFE model, nonlinear relationships in data can be covered more effectively compared to the baseline SAFE model [45]. In another study authors report results of ensemble ANFIS application to countries’ SD performance assessment [30] also using the SAFE dataset [45]. In this case ANFIS was used for discovering fuzzy rules from 128 countries. Furthermore, they trained the ensemble of ANFIS model with various membership functions, which resulted in a more accurate assessment of the overall countries’ SD performance compared to other machine learning methods. Another study [46] was conducted on UCI data, where authors employed ANFIS method for urban sustainability assessment in 185 Chinese cities. After training the model on 740 instances, tested on 185 and validated on 370 instances, they conclude that ANFIS is an appropriate tool to assess urban sustainability. The limitations of these studies are in using the limited datasets, and in the case of ensemble learning there is also a challenge with the computational complexity, which makes it less appropriate for the real time applications. An integration of AHP, fuzzy sets in geographical information systems were used in addressing SD of wheat production in Iran [31]. Furthermore, authors employed ANFIS “to examine whether or not the sustainability indices which are determined by the geographical information system can be estimated” [31]. Authors argue that ANFIS can be a superior alternative tool for sustainability prediction, with the main obstacles identified in that the real data is needed and training should be repeated in certain time intervals. All of the studies that we could find to date address the problem of SD performance assessment and none addresses prediction of the importance of the SDGs and overall importance of the three SD pillars using demographic data. Therefore, the research question, addressing the identified research gap is: “Can importance of the three SD pillars (economic, environmental, and social) be efficiently predicted by demographic data using the ANFIS method?”

## Materials and methods

For the purpose of this study, an online questionnaire was used on a representative sample of young people aged from 14 to 30 years old. The sample size of Serbian youth population has been calculated based on the online sample size calculator [18], taking into consideration the overall number of Serbian youth [19]. The research has been conducted during the September and October 2018, solely for scientific and academic purposes. Participants were informed about the nature of the research and information necessary for their voluntary decision regarding “to” or “not to” participate in the study. Participants who decided to freely and willingly participate in the research study also gave verbal consents. They were assured of anonymity. The research was approved by the Ethical Committee for Research in Organizational Sciences at University of Belgrade—Faculty of Organizational Sciences.

The questionnaire consisted of 28 questions. The first five questions were of general nature–gender, age, place of birth, permanent residence, education. From question 6 to question 11, participants were asked to give their response on following questions: whether they’ve heard of the term sustainable development, and what its definition is; their knowledge of the National Strategy of Sustainable Development of the Republic of Serbia and new goals of sustainable development, as well as of their attitude towards the necessity of active engagement of youth in questions that refer to sustainable development of Serbia. In the following few questions (from question 12 to question 28), the participants of this research have awarded marks for each of the 17 SDGs, having in mind its importance for Serbia and young people. Likert type scale, which ranges from 1 to 5, was used for evaluation, where: 1—the least significant/insignificant, 2 –not so significant, 3 –significant, 4 –very significant, 5 –the most significant.

### Data analysis

An analysis of the results was carried out using the SPSS 24 software package. Descriptive statistics have been used in order to analyze the characteristics of the sample, while ANFIS model was used for forecasting.

For forecasting the marks of certain SD pillars, input data in ANFIS were actually the answers obtained from the questionnaire (gender, age, place of birth, type of settlements, education). In order for these data to be used as input data for the system, all values were previously converted to match adequate numerical values according to:

- Gender (two categories: female and male).
- Age (three categories: from15 to 19 years, from 20 to 24 years and from 25 to 30 years old).
- Types of settlements (two categories: rural and urban).
- Level of education (five categories: elementary school, high school students, college completed, students, faculty completed).

**Fig 1:**
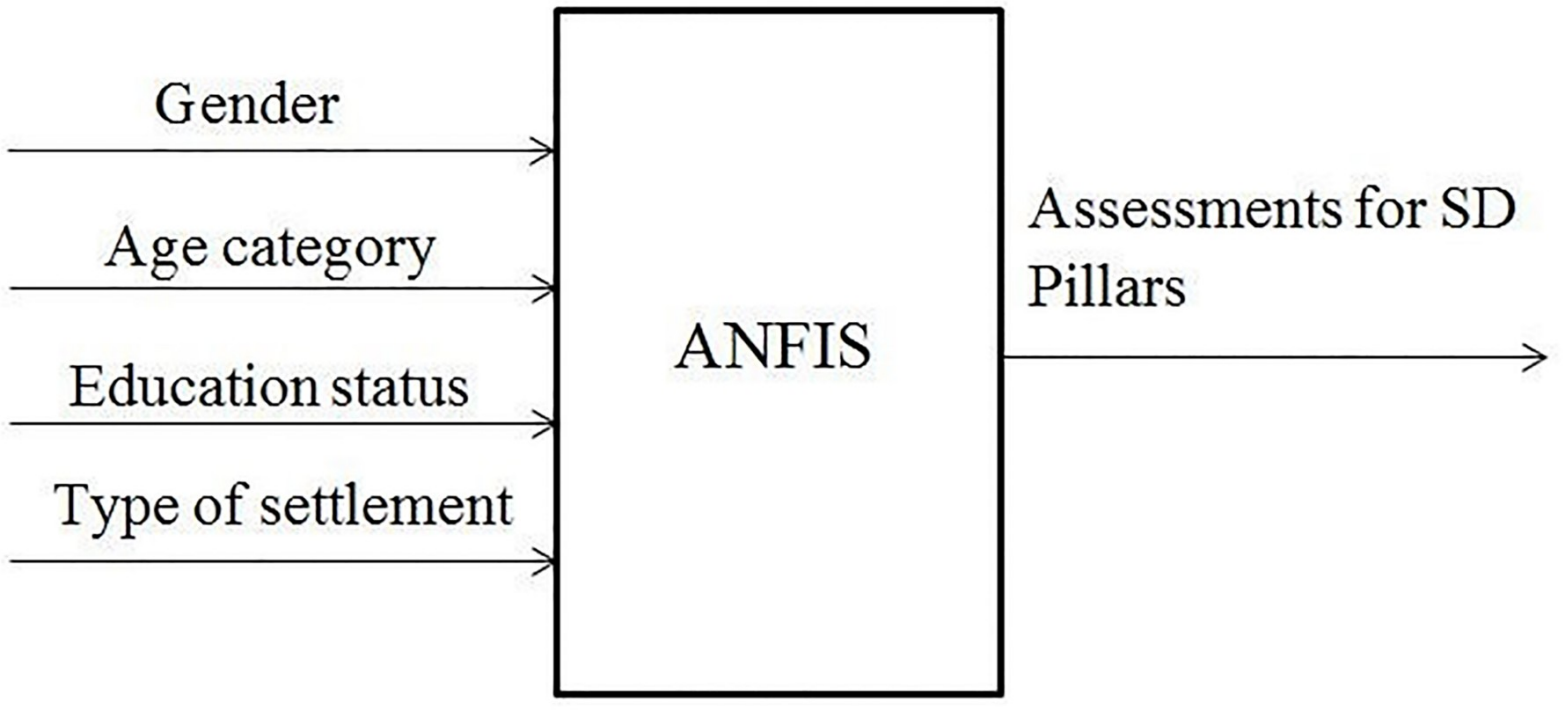
Input and output variables of ANFIS.

As an output from the system, the marks of each of the three SD Pillars have been used. In order to obtain marks for SD Pillars, the mark of the belonging 17 SDGs was calculated for each individual pillar: economic, environmental and social (Fig 1).

For the purposes of this paper, SDGs were integrated into SD Pillars according to authors Cutter, Osborn, Romano and Ullah (2015) and Le Blanc (2015) [47, 48]. The aim of this integration of SDGs is balancing of all three SD pillars within the SDGs framework by [47]:

- Using a systemic, holistic, systems-based approach that reflects the complexity of the existing interconnections in today world.
- Balancing of all three SD pillars within goals and targets.
- Using of exact internal connections between themes and dimensions.
- The proposed integration of SDGs into SD Pillars is given in Table 2.

**Table 2:** Integration of SDGs into SD pillars.

Order	SD Pillars	Belonging SDGs
**1**.	Economic	SD7. Ensure access to affordable, reliable, sustainable and modern energy for all. SD8. Promote inclusive and sustainable economic growth, employment and decent work for all. SD9. Build resilient infrastructure, promote sustainable industrialization and foster innovation. SD12. Ensure sustainable consumption and production patterns. SD16. Promote just, peaceful and inclusive societies. SD17. Revitalize the global partnership for sustainable development.
**2**.	Environmental	SD6. Ensure access to water and sanitation for all. SD7. Ensure access to affordable, reliable, sustainable and modern energy for all. SD12. Ensure sustainable consumption and production patterns. SD13. Take urgent action to combat climate change and its impacts. SD14. Conserve and sustainably use the oceans, seas and marine resources. SD15. Sustainably manage forests, combat desertification, halt and reverse land degradation, halt biodiversity loss.
**3**.	Social	SD1. End poverty in all its forms everywhere. SD2. End hunger, achieve food security and improved nutrition and promote sustainable agriculture. SD3. Ensure healthy lives and promote well-being for all ages. SD4. Ensure inclusive and quality education for all and promote lifelong learning. SD5. Achieve gender equality and empower all women and girls. SD7. Ensure access to affordable, reliable, sustainable and modern energy for all. SD10. Reduce inequality within and among countries. SD11. Make cities inclusive, safe, resilient and sustainable. SD12. Ensure sustainable consumption and production patterns. SD16. Promote just, peaceful and inclusive societies.

What can be seen from Table 2 is that certain SDGs do not belong only to one SD Pillar (e.g. Goal no 12—Ensure sustainable consumption and production patterns, which can fit under all SD Pillars). Contrary to some of the SDGs and their targets are more oriented to one SD Pillar (e.g. Goal no 15—Sustainably manage forests, combat desertification, halt and reverse land degradation, halt biodiversity loss goal on ecosystems, which fit under environmental SD Pillar). “So, it is not to be expected that all three dimensions could have equal weight in every one of the individual goals and its set of targets” [47].

**Fig 2:**
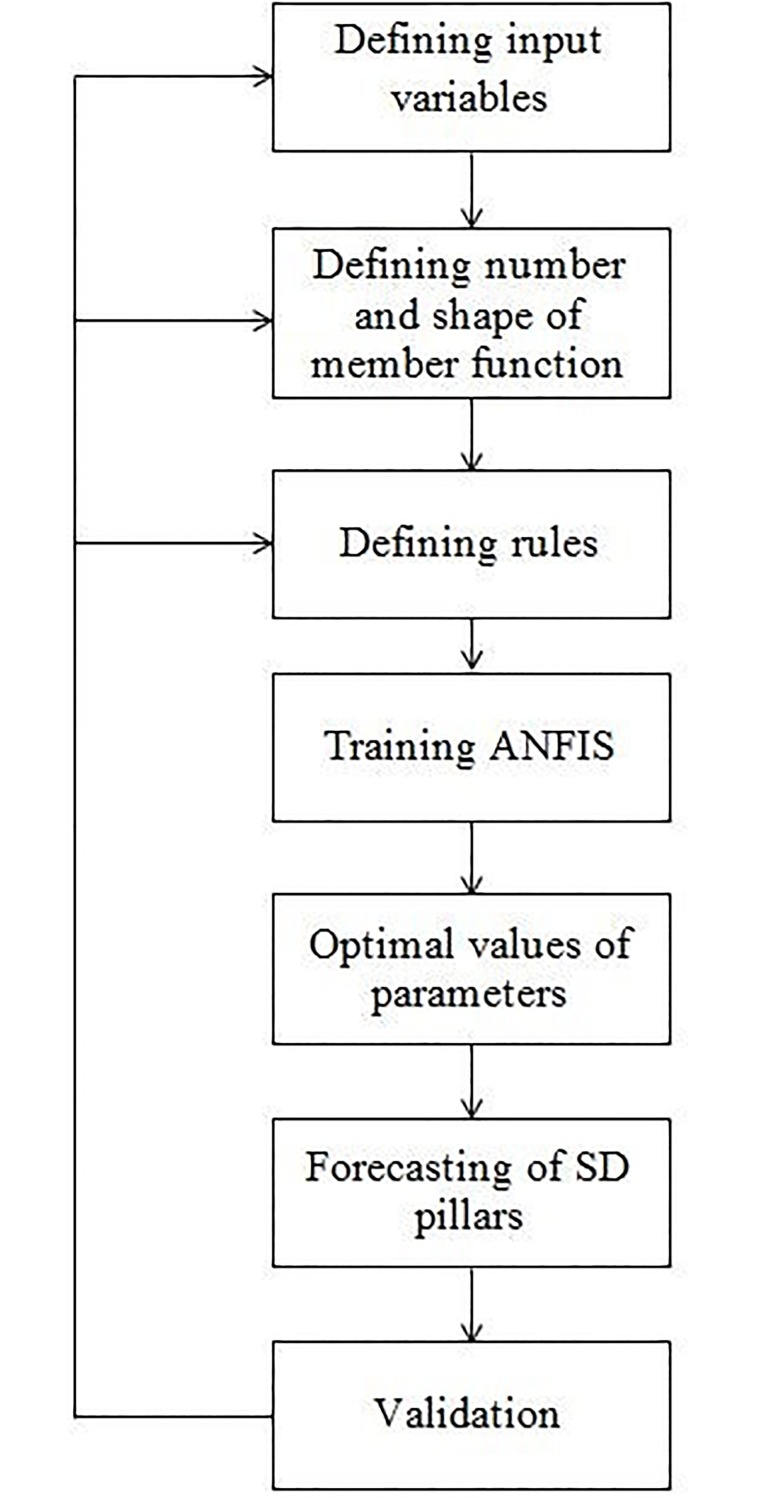
The process of ANFIS development.

A software package MATLAB 2015 has been used for the application of ANFIS. Every input variable in the system is described in over 3 Gauss function. A constant that was most suitable for the needs of the research was used as output variable. The set of 300 input-output data (results obtained from the questionnaire answered by 300 respondents–training set) was used for training the model, while the results of the remaining 86 respondents were used for testing the model. In this way, the suggested application of ANFIS was adequately evaluated. The process of ANFIS development is given in Fig 2.

## Results and discussion

Our research sample included 386 young people, and out of that number:

- 240 of them (62.2 percent) were female,
- 146 of them (37.8 percent) were male.

Age of respondents is the following:

- from 15 to 19 years– 89 of them (23.1 percent),
- from 20 to 24 years– 169 of them (43.8 percent),
- from 25 to 30 years– 128 of them (33.2 percent).

Current status of education of respondents is:

- 76 of them (19.7 percent) completed elementary school,
- 195 of them (50.5 percent) are students of four-year high school,
- 16 of them (4.1 percent) finished upper school,
- 74 of them (19.2 percent) are BSc students,
- 25 of them (6.5 percent) completed faculty (MSc students).

When it comes to the place of living:

- 306 respondents (79.3 percent) are from cities or urban areas,
- 80 of them (20.7 percent) are from small towns or villages.

Based on the assigned ratings and calculated arithmetic mean, 17 SDGs were ranked according to the opinion of young people in relation to their importance not only for Serbia, but for the young as a category as well. The results were presented in Table 3.

**Table 3:** Ranked SDGs by arithmetic mean value.

Order	SDGs	Arithmetic Mean (t_n_)	Standard deviation	Rank
1.	End hunger, achieve food security and improved nutrition and promote sustainable agriculture.	4.72	0.58	1
2.	Ensure access to water and sanitation for all.	4.55	0.71	2
3.	Ensure healthy lives and promote well-being for all ages.	4.42	0.75	3
4.	Sustainably manage forests, combat desertification, halt and reverse land degradation, halt biodiversity loss.	4.41	0.74	4
5.	End poverty in all its forms everywhere	4.27	0.79	5
6.	Ensure inclusive and quality education for all and promote lifelong learning.	4.21	0.73	6
7.	Achieve gender equality and empower all women and girls.	4.17	0.92	7
8.	Promote inclusive and sustainable economic growth, employment and decent work for all.	4.13	0.82	8
9.	Take urgent action to combat climate change and its impacts.	4.12	0.83	9
10.	Ensure access to affordable, reliable, sustainable and modern energy for all.	4.11	0.82	10
11.	Conserve and sustainably use the oceans, seas and marine resources.	3.95	1.05	11
12.	Make cities inclusive, safe, resilient and sustainable.	3.9	0.89	12
13.	Build resilient infrastructure, promote sustainable industrialization and foster innovation.	3.8	0.83	13
14.	Ensure sustainable consumption and production patterns.	3.84	0.95	14
15.	Promote just, peaceful and inclusive societies.	3.82	0.95	15
16.	Reduce inequality within and among countries.	3.78	0.91	16
17.	Revitalize the global partnership for sustainable development	3.68	0.99	17

As it can be seen, the most important SDGs, according to the youth opinion, were: End hunger, achieve food security and improved nutrition and promote sustainable agriculture; Ensure access to water and sanitation for all; Ensure healthy lives and promote well-being for all at all ages with high mean values: 4.72, 4.55 and 4.42.

From the questionnaire it can be observed that the least important SDGs as perceived by the respondents were the following: Promote just, peaceful and inclusive societies; reduce inequality within and among countries; Revitalize the global partnership for sustainable development with mean values of 3.82, 3.78, and 3.68 respectively.

Based on the individual mean values of SDGs and the model proposed in [47, 48] we have calculated the aggregated values of each SD pillar for the whole sample of 386 respondents. The obtained mean values of the economic, environmental, and social pillar as recognized by the respondents are 3.91, 4.16, and 4.124 respectively. This means that as shown by the surveyed youth in Serbia, the most important SD pillar is environmental, followed by the social and least important is the economic SD pillar. The obtained results show that the priority, as observed by the youth, is given to the environment (environmental pillar received the highest values of importance), which is in line with the statement that “environmental protection is the third pillar and, to many, the primary concern of the future of humanity” [49]. On the other hand, it can be a reflection of the bad state of environment in the Republic of Serbia [23, 50] and the need to overcome this state. The importance of social pillar was ranked second. This is an implicit indicator of the youths’ wish for social development, which supports the fact that they need to actively participate in shaping the future in order to continue sustainably [51]. The least important economic SD pillar, as noted by the respondents, come to some surprise knowing that the Republic of Serbia belongs to the poor countries. According to the data relating to economic indicators: gross domestic product (GDP) per capita, and level of actual individual consumption (AIC) per capita, places the Republic of Serbia at the 34^th^ place out of 37^th^ countries, sharing its rank with the Republic of North Macedonia [52]. Furthermore, according to the data of economic field, which comprises of: economic independence of young people, youth unemployment rate, labor force rate, NEET rate, self-employed young people, etc., the Youth Participation Index 2017 for young people from the Republic of Serbia is 5.69, which is very low compared to the EU value of 42.2, the–targeted value according to Agenda 2020 Council of Europe [53]. Furthermore, these results are in line with the results of the research published by the Ministry of youth and sports of Serbia, which shows that only 23.5 percent of the young perceive the unemployment and economic situation as a problem [54].

### Forecasting SD pillars using ANFIS

Various methods, techniques and tools for prediction are presented in the literature such as different type of regressions, neural networks, support vector machines etc. The reason why ANFIS was selected lies in the fact that its structure enables learning directly from data and generating rules in form of natural language. Through mapping input values into membership functions, values of membership functions into the parameters of fuzzy rules, results of individual fuzzy rules into overall output, the ANFIS model can support the decision making process.

The authors of this paper developed and applied a separate ANFIS model for each individual SD pillar (economic, environmental, social), where each had the same structure, i.e. the same number and shape of the membership functions, rules, and the same shape of the output function.

**Fig 3:**
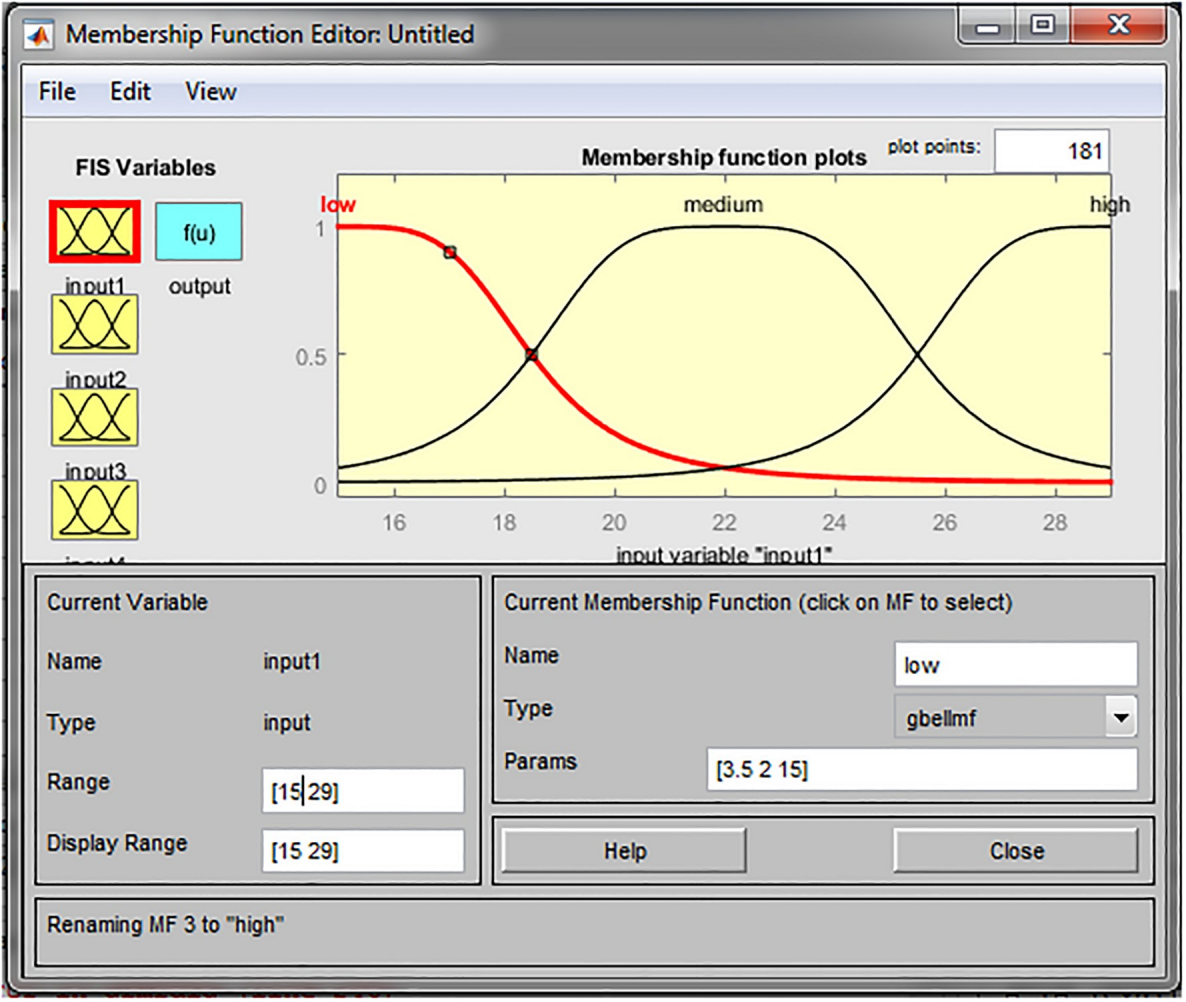
Membership functions for the variable age of respondents.

More precisely, for initial membership functions we used bell-shaped functions. Testing different types of non-linear membership functions, bell-shaped functions have shown to be the most suitable for presenting input variables. Each input is represented by three fuzzy sets i.e. three linguistic variables: low, medium and high. Fig 3 represents the “age of respondents” input variable describes by three fuzzy sets.

**Fig 4:**
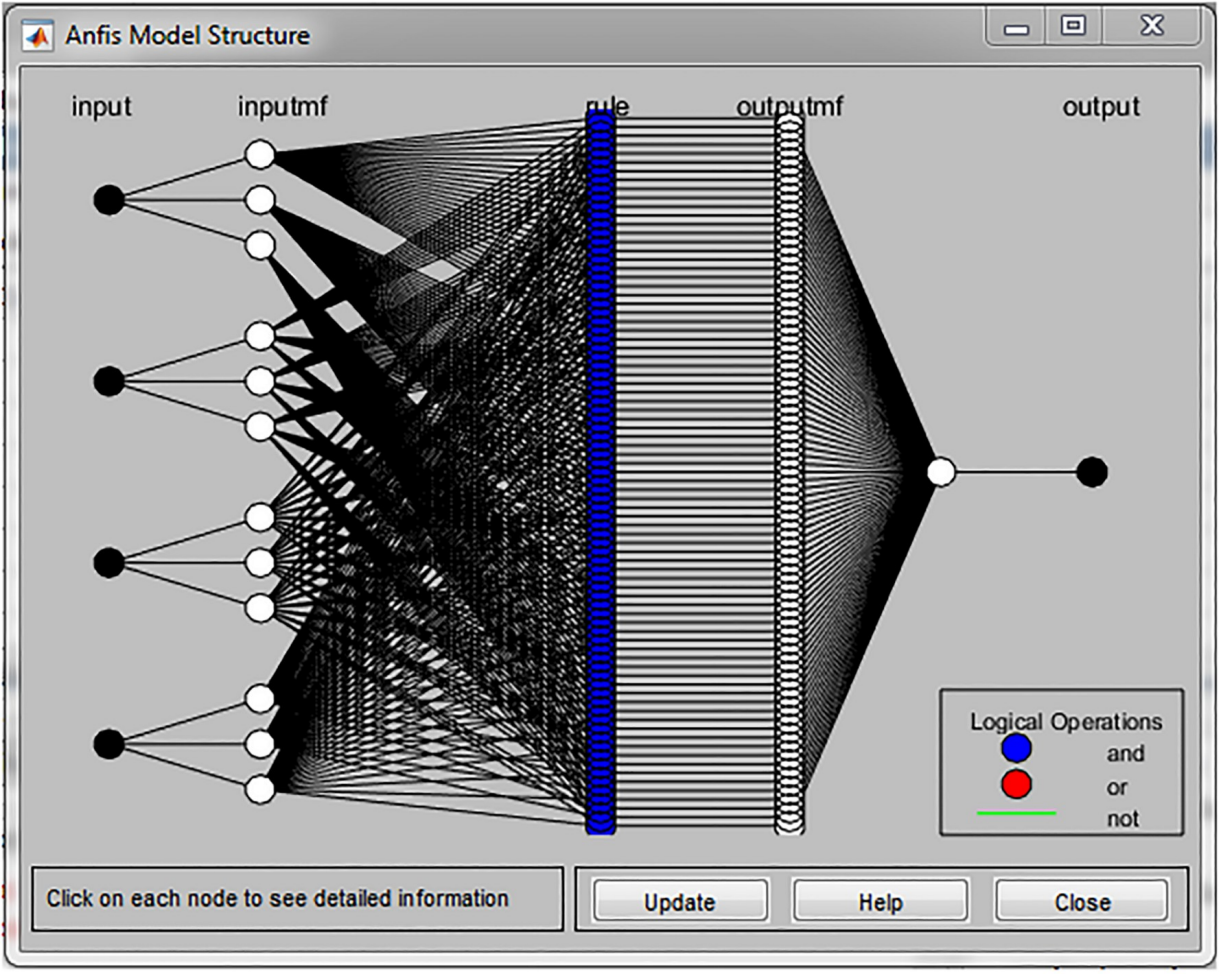
ANFIS model structure for forecasting of SD pillars.

In total 81 if-then rules were generated by MATLAB software, since we used four input variables (gender, age of respondents, current status of education and type of settlement) and each of them is described by three membership functions, as illustrated in Fig 4.

ANFIS is an adaptive system: initial parameters of membership functions are tuned through the learning process, on the basis of input/output set, while its structure and number of layers remain unchanged. Learning process is accomplished by backpropagation gradient method and the least-square method. Optimized parameters of chosen bell-shaped functions are derived from training data rather than expert knowledge, which means less subjectivity. The model was trained on a set of 300 respondents’ answers and tested on the remaining 86 instances. The ratio between training and testing data is 3.4 : 1. Testing data are used for model validation, as measures of goodness of the proposed model for prediction of SD importance.

**Fig 5:**
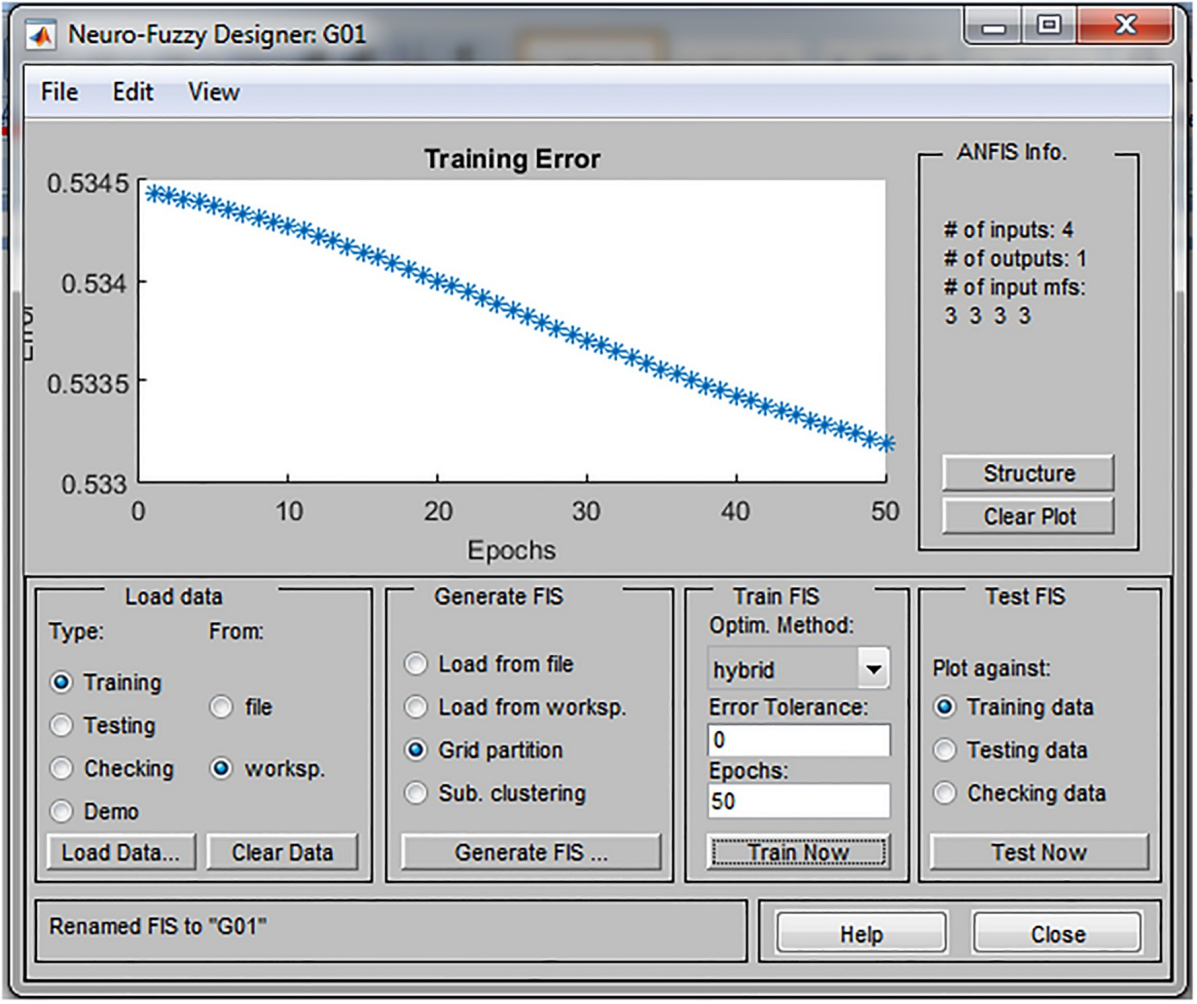
Prediction error of the ANFIS model for environmental pillar.

**Fig 6:**
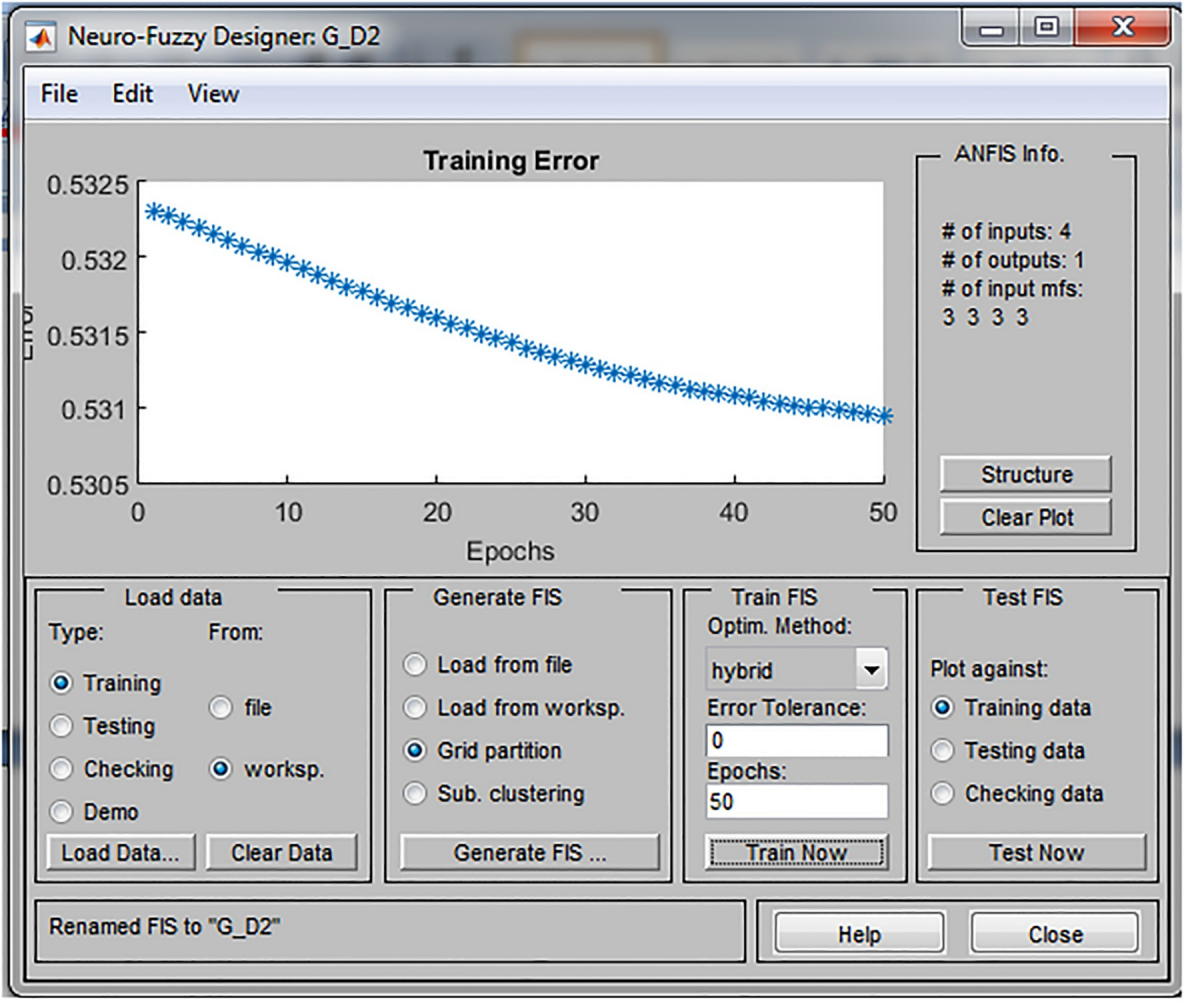
Prediction error of the ANFIS model for economic pillar.

**Fig 7:**
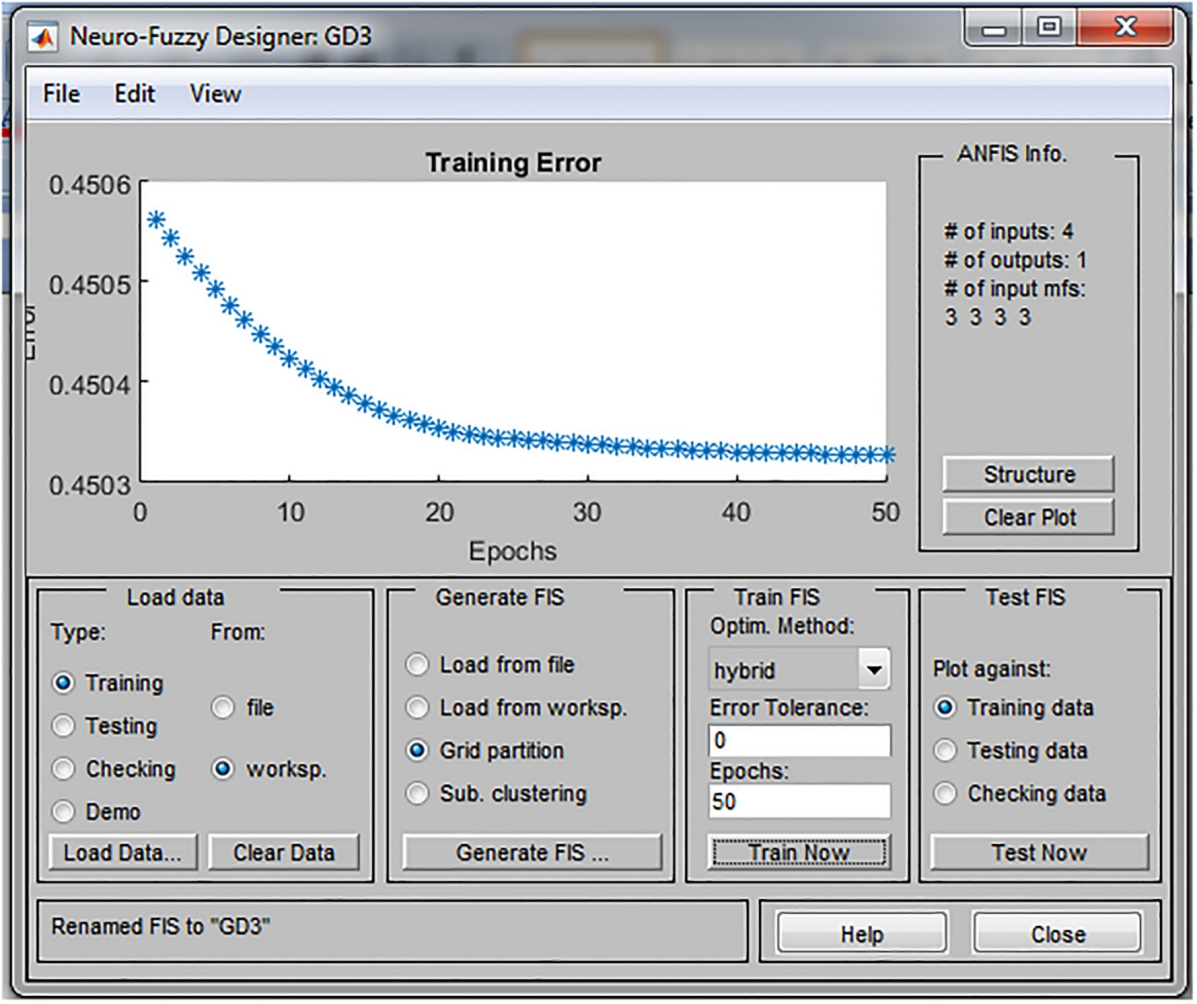
Prediction error of the ANFIS model for social pillar.

Training was conducted in 50 epochs and the training error was in the range between 0.43 and 0.53. Through the learning process, parameters of membership function are tuned in order to be more suitable for SD forecasting. As a consequence, the training error in each individual ANFIS (environmental, economic and social pillar of SD, respectively) decreases as shown in Figs 5–7.

The results of the ANFIS model on a test set:

- The average mark (mean value) of 86 control respondents for the first—economic pillar was 3.47 on the Likert type scale from 1 to 5. The obtained average predicted mark by ANFIS model was 3.63 which means that the relative error of forecasting was 4.61 percent, while the individual absolute error per respondent stood at 0.85.
- The average mark for environmental pillar is 3.92, where the average predicted mark was 4.07, i.e. the relative error of forecasting was 3.83 percent. For this pillar, the average absolute error per respondent is 0.50.
- When it comes to the third, social pillar, the average mark obtained from the respondents was 3.93, and the average forecasted mark 3.92, i.e. relative error was only 0.25 percent, while the average absolute error per respondent was the lowest for this pillar and totaled 0.38.

Tables 4 and 5 display the results obtained in our research for ANFIS model application for the test set of 86 control respondents.

**Table 4:** Results of ANFIS model application in forecasting of SD pillars.

	Economic Pillar	Environmental Pillar	Social Pillar
Actual value of mark	3.47	3.92	3.93
Predicted value of mark by ANFIS model	3.63	4.07	3.92
RelErr	4.61%	3.83%	0.25%
Average absolute error	0.85	0.50	0.38

**Table 5:** Relative error of ANFIS model for SDpillars forecasting.

	Economic Pillar	Environmental Pillar	Social Pillar
RelErr (%)	4.61	3.83	0.25
RelErr <5%	x	x	x
RelErr >5%			

**Fig 8:**
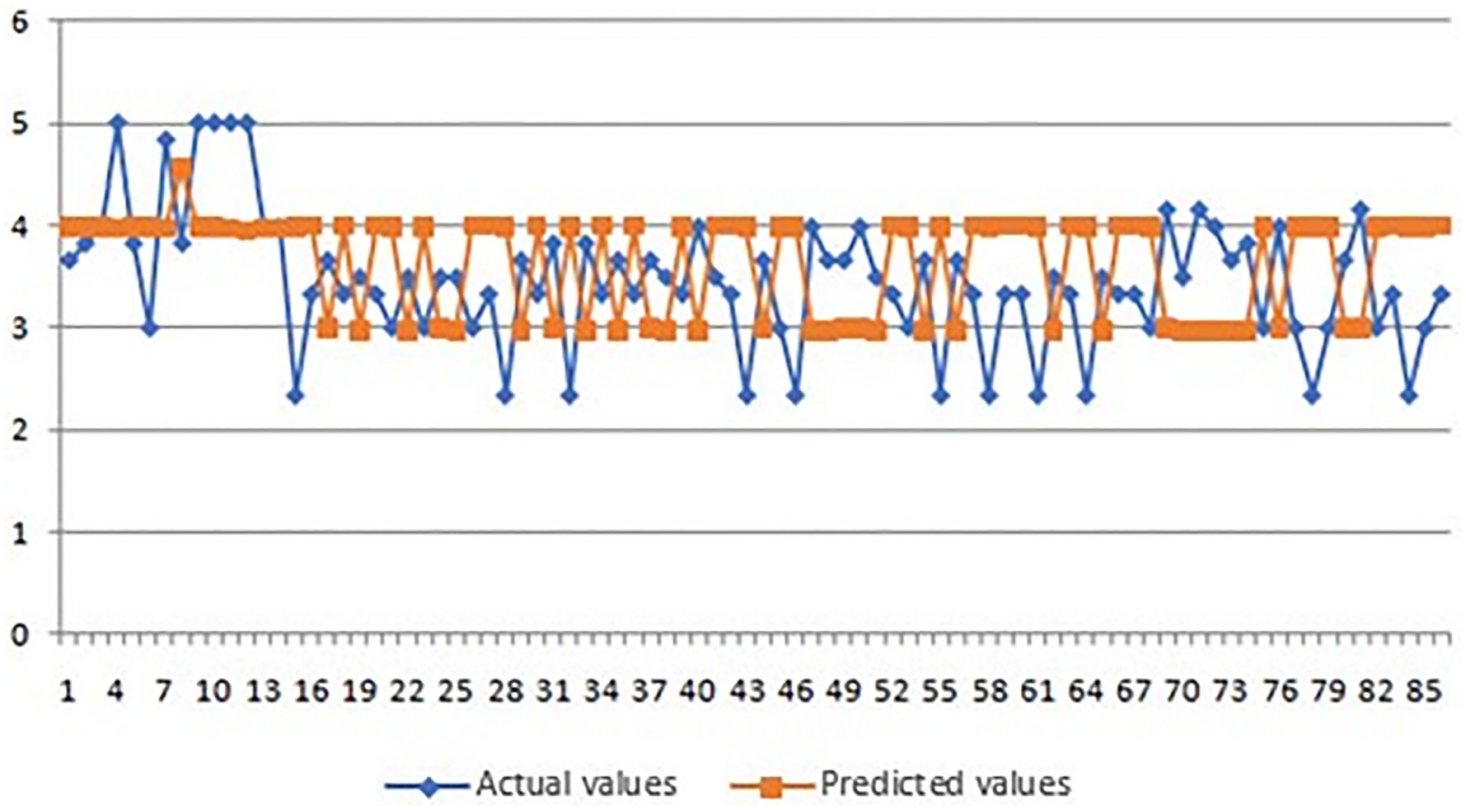
Actual and predicted values of marks for economic pillar.

**Fig 9:**
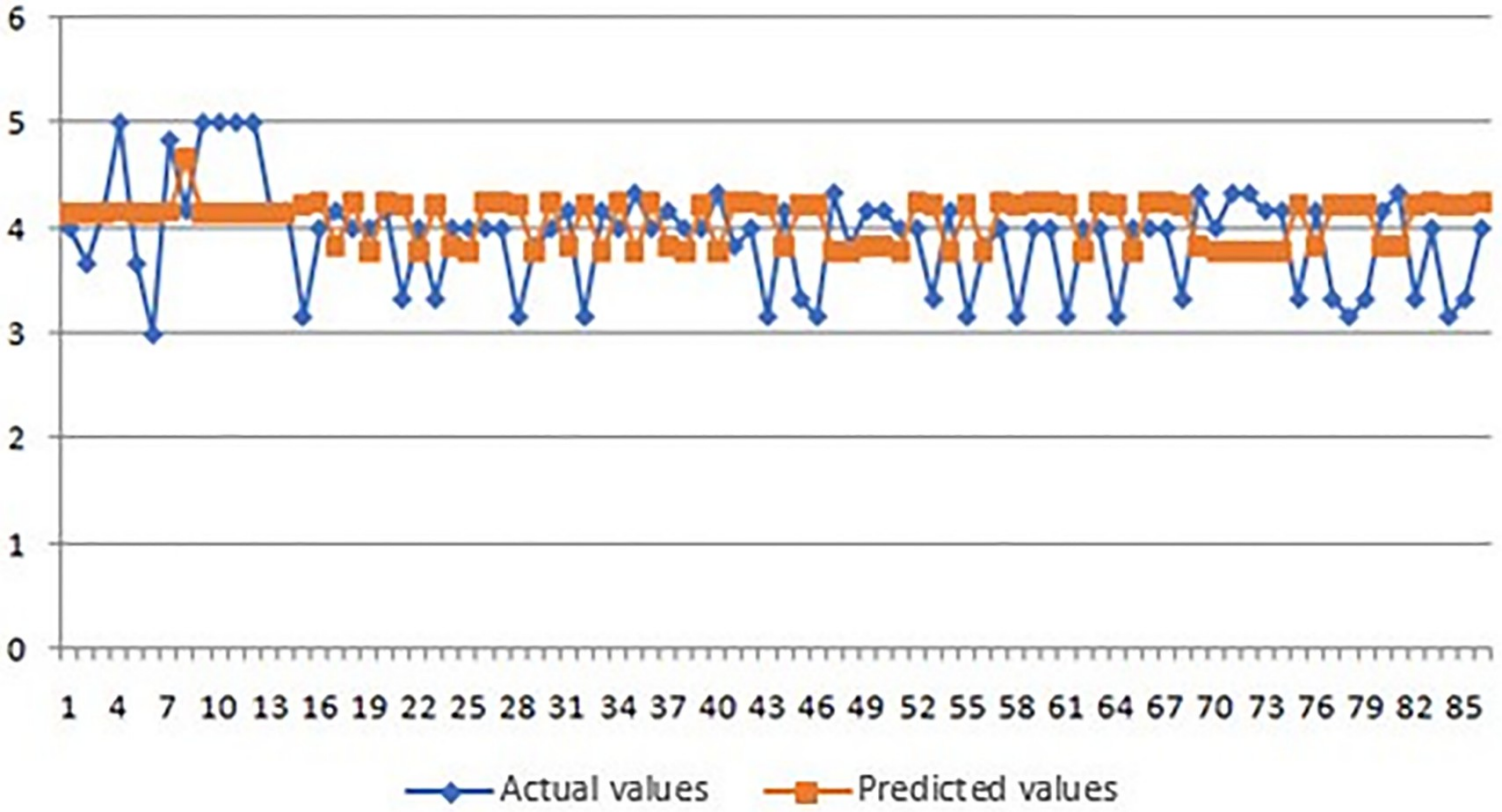
Actual and predicted values of marks for environmental pillar.

**Fig 10:**
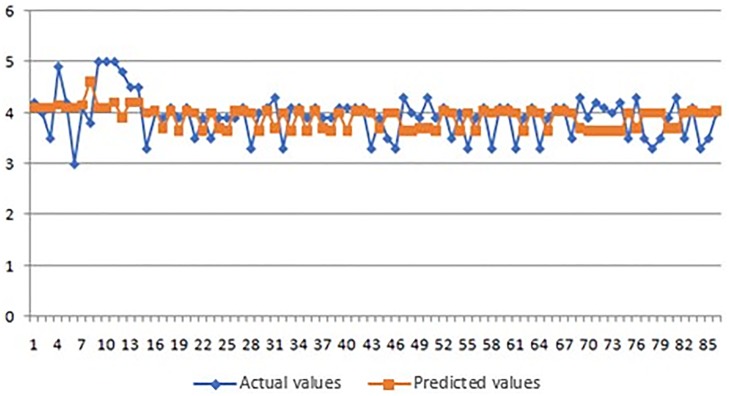
Actual and predicted values of marks for social pillar.

In Figs 8–10 we present graphic displays of the relation of actual and predicted values of marks of the respondents for economic, environmental and social pillar respectively.

As it can be seen from the obtained results of the application of ANFIS model (Tables 3–5 and Figs 8–10), the smallest average error of prediction for all respondents is the one of 0.38 (obtained for social pillar forecasting) which is only 0.25 percent with satisfying relative error of Rel Err < 5%. Also, the adequate difference between the overall obtained and overall forecasted average mark is found in environmental pillar. This difference totals 0.50, which is 3.83 percent with satisfying relative error of Rel Err < 5%. The biggest average error of 0.85 was found with forecasting the mark for economic pillar, with 4.61 percent and with satisfying Rel Err < 5%.

The validation of the proposed ANFIS model was performed by applying the trace-driven simulation test. The model of validation was driven by input derived from real data or trace data [55], which in our study represents data from all 386 respondents.

Tables 6 and 7 display the results obtained for the validation process of ANFIS model application.

**Table 6:** Results of validation of ANFIS model application in forecasting of SD pillars.

	Economic Pillar	Environmental Pillar	Social Pillar
Actual value of mark	3.91	4.16	4.124
Predicted value of mark by ANFIS model validation	3.94	4.20	4.121
Rel Err	1%	1%	0.07%
Average absolute error	0.51	0.44	0.36

**Table 7:** Relative error of validation of ANFIS model for SD pillars forecasting.

	Economic Pillar	Environmental Pillar	Social Pillar
Rel Err (%)	1	1	0.07
Rel Err < 5%	x	x	x
Rel Err > 5%			

**Fig 11:**
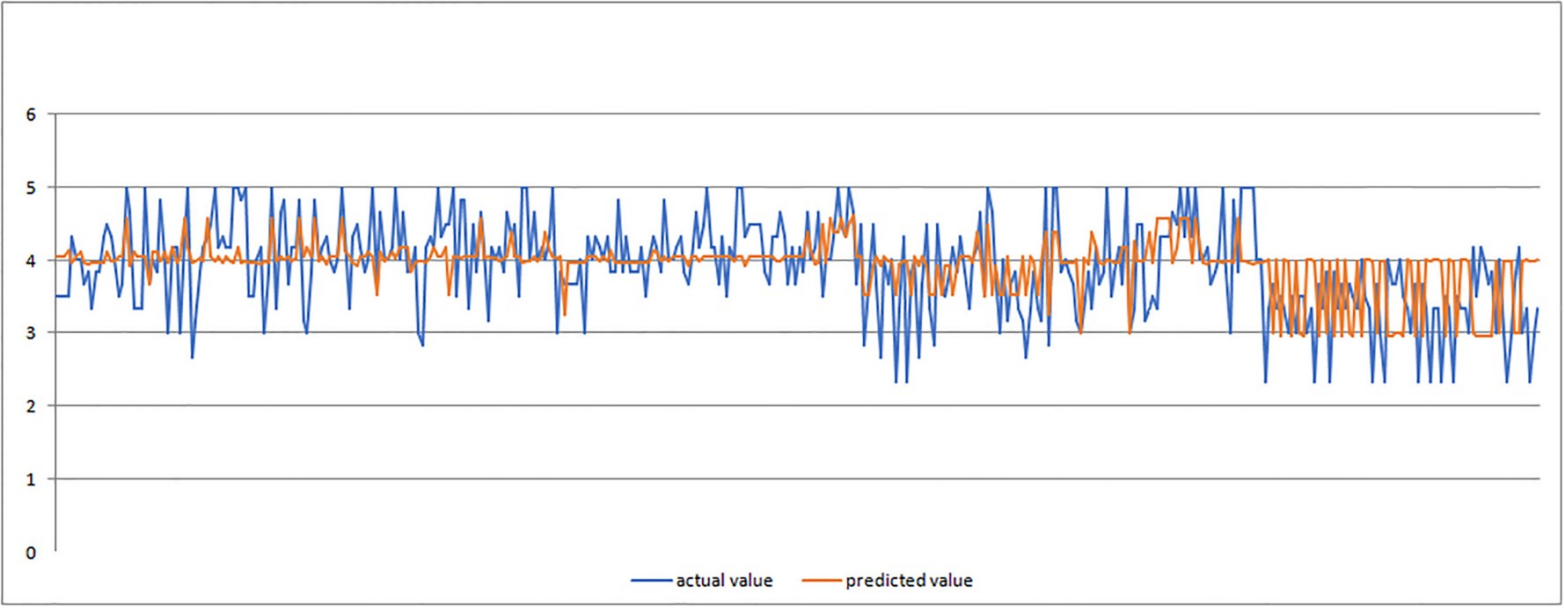
Actual and predicted values of marks for economic pillar (validation of ANFIS model).

**Fig 12:**
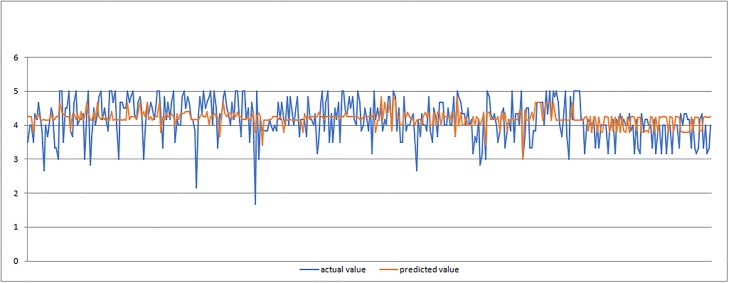
Actual and predicted values of marks for environmental pillar (validation of ANFIS model).

**Fig 13:**
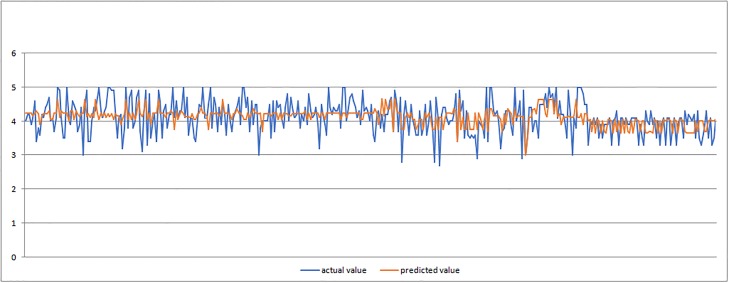
Actual and predicted values of marks for social pillar (validation of ANFIS model).

In Figs 11–13 we present graphic displays of the relation of actual and predicted values of marks of validation of ANFIS model for economic, environmental and social pillar respectively.

The ranked SD pillars by prediction of arithmetic mean value are presented in Table 8.

**Table 8:** Ranked SD pillars by prediction of arithmetic mean value.

Order	SD pillars	Prediction of Arithmetic mean (t_n_)	Rank
**1**.	Environmental pillar	4.20	1
**2**.	Social pillar	4.121	2
**3**.	Economic pillar	3.94	3

The results of the validation process (Tables 6 and 7 and Figs 11–13) clearly demonstrated successful outcomes of application of proposed ANFIS model. Again, the results showed the smallest average error of 0.36 for social pillar forecasting which represents only 0.07 percent with satisfying relative error of Rel Err < 5%. When it comes to environmental pillar, the average mark is 0.44, which is 1percent with satisfying relative error of Rel Err < 5%. The average error of 0.51 was found with forecasting the mark for economic pillar, which is 1percent with satisfying Rel Err < 5%. It must be noted that the validation process performed well and confirmed good applicability of proposed ANFIS model application.

Based on the obtained results, it can be concluded that the authors of the paper were using sufficient and adequate defined data (input variables, number and shape of membership function, and rules) for inputs into ANFIS model with satisfying relative error of Rel Err < 5% in the forecasting of the system output, i.e. the assessment of the importance of all three SD pillars. This means that ANFIS is capable of capturing nonlinear relationship between input variables and outputs. Having in mind that this is a pilot study, and that in this phase of research the applied system of forecasting did not take into consideration all possible inputs for more comprehensive research, in future studies, more attention should be paid to the large datasets and the increase in training set which makes it easier to come to optimal parameters of the obtained system. In addition, our future research can combine other neuro-fuzzy methods with ANFIS for the forecasting of SD pillars.

## Conclusions

The young represent the key stakeholders and participants in all the questions that refer to SD and viability, and “has the right of a sustainable future and the right of being part of this transformational process” [56]. They play necessary active role in the adequate decision making processes that relate to integration of all SDGs as well as to each individual SD pillar–economic, social and environmental [57–60]. Therefore, the opinion of young people must be taken into consideration as a necessary element of SD, since their involvement in the matters of sustainability is important for more successful and better functioning of every society [4]. This also points out to the fact that all research that refer to the question of young people and SD contributes to necessary body of knowledge of this crucial issue, having in mind that: “The creativity, ideals and courage of the youth of the world should be mobilized to forge a global partnership in order to achieve sustainable development and ensure a better future for all” [3].

Furthermore, to our knowledge an Adaptive Neuro-Fuzzy Inference System–ANFIS has never been applied for forecasting of all three pillars of SD [25–32].

The research was carried out on a representative sample of young people of Serbia consisting of 386 respondents, out of which 300 was used as a training set, and the remaining 86 for testing the model.

The obtained results included not only the ranking of the importance of SDGs (End hunger, achieve food security and improved nutrition and promote sustainable agriculture–rank 1; Ensure access to water and sanitation for all–rank 2; Ensure healthy lives and promote well-being for all ages–rank 3) but also the predictions with the satisfying accuracy of all three SD pillars (environmental, social, economic): with marks 4.20, 4.121, and 3.94 respectively, and the relative error of < 5%.

This study clearly indicates that the applied ANFIS method based on ‘only’ four input variables (gender, age of respondents, current status of education and type of settlement) provides an acceptable predictions of SDG importance. Optimization of the input and output parameters, questionnaire and adequate selection of the research sample, is capable of providing sufficiently good forecasting of the three pillars of SD.

Such predictions cannot replace the discussion with the young, but can help steering the discussion in directions that are important to the young people in question. By knowing and understanding what is important to the youth, we can gain their trust and achieve better collaboration in creating SD policies. To our knowledge this is the first application of ANFIS method in forecasting the importance of SDGs from demographic data. Nevertheless, we have to be aware that the ground truth can change in time, a phenomenon that in machine learning is called a concept drift, that is why we should keep updating the model in order not to miss an important change.

In the end, we can draw a conclusion that ANFIS model applied in our study can be used in future research (with the increase in the number of respondents and larger training set, as well as with the application of other methods of artificial intelligence and comparative analysis of obtained results and different normalization methods for input variables) in the development of youth policies, local action and environmental plans, and adequate application and development of national strategies and SD policies.

## Supporting information

S1 File Input data from questionnaire. (XLS)

S2 File ANFIS model. (XLS)

S3 File Validation of ANFIS model. (XLS)

S4 File Questionnaire youth and SDGs (Serbian). (DOC)

S5 File Questionnaire youth and SDGs (English). (DOC)
