# Supplementary material for: Youth and forecasting of sustainable development pillars: An adaptive neuro-fuzzy inference system approach
Source: PLoS One. 2019 Jun 25;14(6):e0218855. doi: 10.1371/journal.pone.0218855 (PMC6592548; doi:10.1371/journal.pone.0218855)
Supplement: S4 File — (DOC) [file pone.0218855.s004.doc]

***Upitnik***

**MLADI I CILJEVI ODRŽIVOG RAZVOJA U SRBIJI**

Hvala vam što ste dobrovoljno pristali da učestvujete u upitniku koji se odnosi na ciljeve održivog razvoja, a koje je odborio Etički komitet za istraživanje u organizacionim naukama Univerziteta u Beogradu – Fakulteta organizacionih nauka.

Upitnik predstavlja sastavni deo stručnog monitoringa Centra za ekološki menadžment i održivi razvoj Univerziteta u Beogradu - Fakulteta organizacionih nauka, Srbija, a njegovi rezultati biće iskorišćeni isključivo u naučne i akademske svrhe.

U upitniku, ćete biti pitani da rangirate postojeće ciljeve održivog razvoja preciznije da im dodelite ocene koje po vašem mišljenju predstavljaju njihovu važnost za vas kao mlade u Srbiji.

Vaše učešće u ovom upitniku, kao i vaši pojedinačni odgovori će biti anonimni i strogo poverljivi i dostupni samo istraživačkom timu.

1. Pol

- Ženski
- Muški

1. Koliko imate godina? _________________
2. Mesto rođenja?_____________________
3. Mesto stanovanja:
   - Urbana sredina:_________________
   - Ruralna sredina:_________________
4. Nivo obrazovanja:
   - Zavšena osnovna škola
   - Učenik srednje škole

- Završena viša škola
- Student
- Završen fakultet

1. Da li ste čuli za pojam održivi razvoj?

- Da
- Ne
- Ne znam

1. Ako je na vaše prethodno pitanja odgovor DA, definišite održivi razvoj.

__________________________________________________________________________________________________________________________________________________________________________________

1. Da li u Srbiji postoji Nacionalna strategija održivog razvoja?

- Da
- Ne
- Ne znam

1. Da li ste upoznati sa ciljevima održivog razvoja?

- Da
- Ne

1. Da li mladi treba da učestvuju u pitanjima koja se odnose na održvi razvoj u Srbiji?

- Da
- Ne
- Ne znam

1. Da li mladi učestvuju u pitanjima koja se odnose na održvi razvoj u Srbiji (realna situacija)?

- Da
- Ne
- Ne znam

Rangirajte ciljeve održivog razvoja prema njihovoj važnosti za Srbiju: 1 – *APSOLUTNO NE*, 2 – *NE SLAŽEM SE*, 3 – *NE ZNAM*, 4 – *SLAŽEM SE*, 5 – *APSOLUTNO DA*

| R.br. | CILJ ODRŽIVOG RAZVOJA | Važnost | | | | |
| --- | --- | --- | --- | --- | --- | --- |
| 12. | OKONČANJE SIROMAŠTVA U SVIM NJEGOVIM OBLICIMA SVUDA U SVETU | 1 | 2 | 3 | 4 | 5 |
| 13. | OKONČANJE GLADI, OBEZBEĐIVANJE SIGURNOSTI HRANE, POBOLJŠANJE ISHRANE I PROMOCIJA ODRŽIVE POLJOPRIVREDE | 1 | 2 | 3 | 4 | 5 |
| 14. | OBEZBEĐIVANJE ZDRAVOG ŽIVOTA I PROMOCIJA BLAGOSTANJA ZA SVE U SVIM STAROSNIM DOBIMA | 1 | 2 | 3 | 4 | 5 |
| 15. | OBEZBEĐIVANJE INKLUZIVNOG I KVALITETNOG OBRAZOVANJA I PROMOCIJA CELOŽIVOTNOG UČENJA | 1 | 2 | 3 | 4 | 5 |
| 16. | DOSTIZANJE JEDNAKOSTI POLOVA I JAČANJE SVIH ŽENA I DEVOJAKA | 1 | 2 | 3 | 4 | 5 |
| 17. | OBEZBEĐIVANJE PRISTUPA VODI I SANITARNIM USLOVIMA ZA SVE | 1 | 2 | 3 | 4 | 5 |
| 18. | OSIGURANJE PRISTUPA DOSTUPNOJ, POUZDANOJ, ODRŽIVOJ I MODERNOJ ENERGIJI ZA SVE | 1 | 2 | 3 | 4 | 5 |
| 19. | PROMOCIJA INKLUZIVNOG I ODRŽIVOG EKONOMSKOG RASTA, ZAPOŠLJAVANJA I PRISTOJNOG RADA ZA SVE | 1 | 2 | 3 | 4 | 5 |
| 20. | IZGRADNJA PRILAGODLJIVE INFRASTRUKTURE, PROMOCIJA ODRŽIVE INDUSTRIJALIZACIJE I PODSTICANJE INOVACIJA | 1 | 2 | 3 | 4 | 5 |
| 21. | SMANJENJE NEJEDNAKOSTI UNUTAR I IZMEĐU ZEMALJA | 1 | 2 | 3 | 4 | 5 |
| 22. | OBEZBEĐIVANJE INKLUZIVNOSTI, BEZBEDNOSTI, PRILAGODLJIVOSTI I ODRŽIVOSTI GRADOVA | 1 | 2 | 3 | 4 | 5 |
| 23. | OBEZBEĐIVANJE OBRAZACA ODRŽIVE POTROŠNJE I PROIZVODNJE | 1 | 2 | 3 | 4 | 5 |
| 24. | PREDUZIMANJE HITNIH AKCIJA U BORBI PROTIV KLIMATSKIH PROMENA I NJENIH UTICAJA | 1 | 2 | 3 | 4 | 5 |
| 25. | KONZERVACIJA I ODRŽIVA UPOTREBA OKEANA, MORA I MORSKIH IZVORA | 1 | 2 | 3 | 4 | 5 |
| 26. | ODRŽIVO UPRAVLJANJE ŠUMAMA, BORBA PROTIV DEZERTIFIKACIJE, ZAUSTAVLJANJE I MENJANJE PRAVCA DEGRADACIJE ZEMLJIŠTA, ZAUSTAVLJANJE GUBITKA BIODIVERZITETA | 1 | 2 | 3 | 4 | 5 |
| 27. | PROMOCIJA PRAVDE, MIRA I INKLUZIVNIH DRUŠTAVA | 1 | 2 | 3 | 4 | 5 |
| 28. | REVITALIZACIJA GLOBALNOG PARTNERSTVA ZA ODRŽIVI RAZVOJ | 1 | 2 | 3 | 4 | 5 |

**HVALA ŠTO STE POPUNILI UPITNIK!**
