# Supplementary material for: Youth and forecasting of sustainable development pillars: An adaptive neuro-fuzzy inference system approach
Source: PLoS One. 2019 Jun 25;14(6):e0218855. doi: 10.1371/journal.pone.0218855 (PMC6592548; doi:10.1371/journal.pone.0218855)
Supplement: S5 File — (DOC) [file pone.0218855.s005.doc]

***Questionnaire***

**YOUTH AND SUSTAINABLE DEVELOPMENT GOALS IN SERBIA**

Thank you for voluntarily agreeing to participate in a questionnaire relating to the goals of sustainable development, which was approved by the Ethical Committee for Research in Organizational Sciences at University of Belgrade - Faculty of Organizational Sciences.

The questionnaire is a part of the professional monitoring process of the Center for Environmental Management and Sustainable Development of the University of Belgrade - Faculty of Organizational Sciences, Serbia, and its results will be used exclusively for scientific and academic purposes.

In the questionnaire, you will be asked to rank sustainable development goals and assign them with marks relating to their importance for you as youth in Serbia.

Your participation in this questionnaire, as well as your individual answers will be anonymous, strictly confidential and available only to the research team.

1. Gender

- Female
- Male

1. How old are you? _________________
2. Place of birth?_____________________
3. Types of settlements:
   - Urban:_________________
   - Rural:_________________
4. Level of education:
   - Elementary school
   - High school students

- College completed
- Student
- Faculty completed

1. Have you ever heard of the term sustainable development?

- Yes
- No
- I don’t know

1. If your answer to the previous question is yes, please define the term sustainable development.

__________________________________________________________________________________________________________________________________________________________________________________

1. Does Serbia have the National Strategy for Sustainab Development?

- Yes
- No
- I don’t know

1. Are you familiar with sustainable development goals?

- Yes
- No

1. In your opinion, do young people need to participate in the issues regarding to sustainable development in Serbia?

- Yes
- No
- I don’t know

1. Do young people play a role in questions connected with sustainable development in Serbia (real situation)?

- Yes
- No
- I don’t know

Please rank sustainable development goals according to their importance for Serbia and young people: 1 – *the least significant/insignificant*, 2 – *not so significant*, 3 – *significant*, 4 – *very significant*, 5 – *the most significant*

| No. | SUSTAINABLE DEVELOPMENT GOAL | Importance | | | | |
| --- | --- | --- | --- | --- | --- | --- |
| 12. | END POVERTY IN ALL ITS FORMS EVERYWHERE | 1 | 2 | 3 | 4 | 5 |
| 13. | END HUNGER, ACHIEVE FOOD SECURITY AND IMPROVED NUTRITION AND PROMOTE SUSTAINABLE AGRICULTURE | 1 | 2 | 3 | 4 | 5 |
| 14. | ENSURE HEALTHY LIVES AND PROMOTE WELL-BEING FOR ALL AT ALL AGES | 1 | 2 | 3 | 4 | 5 |
| 15. | ENSURE INCLUSIVE AND QUALITY EDUCATION FOR ALL AND PROMOTE LIFELONG LEARNING | 1 | 2 | 3 | 4 | 5 |
| 16. | ACHIEVE GENDER EQUALITY AND EMPOWER ALL WOMEN AND GIRLS | 1 | 2 | 3 | 4 | 5 |
| 17. | ENSURE ACCESS TO WATER AND SANITATION FOR ALL | 1 | 2 | 3 | 4 | 5 |
| 18. | ENSURE ACCESS TO AFFORDABLE, RELIABLE, SUSTAINABLE AND MODERN ENERGY FOR ALL | 1 | 2 | 3 | 4 | 5 |
| 19. | PROMOTE INCLUSIVE AND SUSTAINABLE ECONOMIC GROWTH, EMPLOYMENT AND DECENT WORK FOR ALL | 1 | 2 | 3 | 4 | 5 |
| 20. | BUILD RESILIENT INFRASTRUCTURE, PROMOTE SUSTAINABLE INDUSTRIALIZATION AND FOSTER INNOVATION | 1 | 2 | 3 | 4 | 5 |
| 21. | REDUCE INEQUALITY WITHIN AND AMONG COUNTRIES | 1 | 2 | 3 | 4 | 5 |
| 22. | MAKE CITIES INCLUSIVE, SAFE, RESILIENT AND SUSTAINABLE | 1 | 2 | 3 | 4 | 5 |
| 23. | ENSURE SUSTAINABLE CONSUMPTION AND PRODUCTION PATTERNS | 1 | 2 | 3 | 4 | 5 |
| 24. | TAKE URGENT ACTION TO COMBAT CLIMATE CHANGE AND ITS IMPACTS | 1 | 2 | 3 | 4 | 5 |
| 25. | CONSERVE AND SUSTAINABLY USE THE OCEANS, SEAS AND MARINE RESOURCES | 1 | 2 | 3 | 4 | 5 |
| 26. | SUSTAINABLY MANAGE FORESTS, COMBAT DESERTIFICATION, HALT AND REVERSE LAND DEGRADATION, HALT BIODIVERSITY LOSS | 1 | 2 | 3 | 4 | 5 |
| 27. | PROMOTE JUST, PEACEFUL AND INCLUSIVE SOCIETIES | 1 | 2 | 3 | 4 | 5 |
| 28. | REVITALIZE THE GLOBAL PARTNERSHIP FOR SUSTAINABLE DEVELOPMENT | 1 | 2 | 3 | 4 | 5 |

**THANK YOU FOR FILLING OUT THIS QUSTIONNAIRE!**
